# Histone modifications associated with herpes simplex virus type 1 genomes during quiescence and following ICP0-mediated de-repression

**DOI:** 10.1099/vir.0.83272-0

**Published:** 2008-01

**Authors:** Heather M. Coleman, Viv Connor, Zara S. C. Cheng, Finn Grey, Chris M. Preston, Stacey Efstathiou

**Affiliations:** 1 Division of Virology, Department of Pathology, University of Cambridge, Tennis Court Road, Cambridge CB2 1QP, UK; 2 Medical Research Council Virology Unit, Glasgow G11 5JR, UK

## Abstract

In the current study, it was shown that repressed virus genomes in quiescently infected MRC5 cells adopt a repressed histone-associated structure marked by the enrichment of deacetylated histones at a wide variety of herpes simplex virus type 1 (HSV-1) promoters. In addition, it was shown that genome de-repression, mediated by HSV-2 superinfection or delivery of ICP0 using a recombinant adenovirus vector, resulted in the enrichment of acetylated histones on HSV DNA. These data indicate that ICP0-mediated genome de-repression is intimately linked to enrichment of acetylated histones at virus promoters. The fold change in association of pan-acetylated histone H3 following Ad.TRE.ICP0-mediated de-repression consistently revealed promoter-specific variation, with the highest fold changes (>50-fold) being observed at the latency-associated transcript promoter and enhancer regions. Chromatin immunoprecipitation analyses using an antibody specific to the C terminus of histone H3 as a surrogate measure of nucleosome occupancy revealed little variability in the total loading of histone H3 at the various HSV promoters. This observation suggests that acetylation of histone H3 in response to ICP0 expression is not uniformly targeted across the HSV-1 genome during ICP0-mediated de-repression.

## INTRODUCTION

Herpes simplex virus (HSV) undergoes productive infection in epithelial cells leading to access of virus to nerve terminals and establishment of latency in sensory neurones. During latency, the virus genome adopts a non-linear configuration and is transcriptionally repressed with the exception of a region encoding the latency-associated transcripts (LATs). The mechanism by which virus genomes are maintained in a repressed state during latency and the processes involved in reactivation from latency remain poorly understood. None the less, there is compelling evidence that histones play an important role in the regulation of HSV gene expression during latency. Analyses of murine brainstem tissue first demonstrated the nucleosomal organization of latent virus DNA (Deshmane & Fraser, 1989), and studies of virus reactivation in neuronal cultures revealed that the immediate-early (IE) ICP0 promoter is activated by the histone deacetylase inhibitor trichostatin A (TSA) (Arthur *et al.*, 2001), implying that latent genomes can respond to changes in the acetylation status of histones.

It has been established that post-translational modifications of the N-terminal tails of histones are involved in the regulation of transcription (Jenuwein & Allis, 2001; Kouzarides, 2002). Thus, hyperacetylation of histones is associated with an ‘open chromatin’ conformation and transcriptional activity, whilst histone hypoacetylation is associated with condensed chromatin and gene silencing. Recent work on HSV-1 suggests that chromatinization of the virus genome and certain accompanying histone modifications offer a means of regulating virus gene expression during lytic infection (Herrera & Triezenberg, 2004; Kent *et al.*, 2004). In the context of latency, it is of significance that studies utilizing chromatin immunoprecipitation (ChIP) assays have shown the LAT promoter to be enriched with acetylated histone H3, whilst representative lytic cycle promoters exhibit a decreased association with acetylated histones (Kubat *et al.*, 2004a, b). The demonstration that acetylated histones are enriched on the ICP0 promoter following the application of a ganglionic reactivation stimulus supports the view that genome de-repression is linked to the acetylation status of histones positioned on lytic cycle promoters (Amelio *et al.*, 2006). Furthermore, data showing that a LAT-deficient mutant exhibits enrichment of histone modifications associated with transcriptional activation suggests that virus functions expressed during latency facilitate maintenance of a repressed genome (Wang *et al.*, 2005).

In order to facilitate studies of histones associated with transcriptionally repressed HSV-1 genomes, we have utilized a fibroblast model of latency based on previously described systems (Harris & Preston, 1991; Preston & Nicholl, 1997; Samaniego *et al.*, 1998). In these systems, replication-defective mutants of HSV-1 are used to infect tissue culture cells and establish a quiescent infection. Repressed virus genomes are retained in a non-linear configuration and, although resistant to a variety of reactivation stimuli, they can be efficiently de-repressed by superinfection with HSV or provision of ICP0 *in trans* (Harris *et al.*, 1989; Zhu *et al.*, 1990; Preston & Nicholl, 1997; Hobbs *et al.*, 2001). These studies and the fact that ICP0 null mutants exhibit reactivation deficits in animal model systems (Leib *et al.*, 1989; Halford & Schaffer, 2001) has led to interest in the properties and function of ICP0 that may be of relevance to the process of reactivation from latency (reviewed by Everett, 2000; Hagglund & Roizman, 2004; Efstathiou & Preston, 2005). In the current study, we show that virus genomes in quiescently infected MRC-5 cells adopted a repressed histone-associated structure marked by the enrichment of deacetylated histones and histone H3 lysine 9 (K9) methylation at a variety of HSV-1 promoters. In addition, we report that genome de-repression mediated by virus superinfection or delivery of ICP0 using a recombinant adenovirus resulted in the enrichment of acetylated histones on HSV DNA. These data indicate that ICP0-mediated genome de-repression is intimately linked to enrichment of acetylated histones at virus promoters.

## METHODS

### Viruses and cells.

MRC-5 cells were maintained in Dulbecco's modified Eagle's medium supplemented with 10 % fetal calf serum (FCS) and 2 mM l-glutamine. Baby hamster kidney (BHK) cells were cultured in Glasgow minimum essential medium supplemented with 10 % FCS, 10 % tryptose phosphate broth and 2 mM l-glutamine. HSV-1 *in*1382 (Homer *et al.*, 1999) contains a 12 bp insertion within VP16 abolishing its transactivation function (Ace *et al.*, 1989), a deletion within the RING finger of ICP0 and a *ts* mutation within ICP4. This replication-defective mutant contains the human cytomegalovirus (HCMV) major IE promoter (−750 to +7) driving expression of *β*-galactosidase inserted at the thymidine kinase gene. *In*1383 is identical to *in*1382 but carries an ICP0 promoter (−850 to +48) *β*-galactosidase cassette. *In*1382 and *in*1383 stocks were produced using BHK cells at 31 °C in the presence of 3 mM hexamethylene bisacetamide as described previously (McFarlane *et al.*, 1992). dl1403 is an ICP0 deletion mutant derived from HSV strain 17 (Stow & Stow, 1986).

### Establishment of quiescently infected MRC-5 cells.

MRC-5 cells were infected with *in*1382 or *in*1383 at an m.o.i. of 1 and incubated at the non-permissive temperature of 39 °C. At 5–6 days post-infection (p.i.), cells were harvested for ChIP assays of repressed virus genomes or induced to reactivate by superinfection with HSV-2 strain 333. Prior to superinfection, cells were incubated for 1 h with 100 μg phosphonoacetic acid (PAA) ml^−1^ and then superinfected with 5 p.f.u. HSV-2 per cell in the presence of PAA. Adenovirus vectors used in this study were kindly provided by Dr Anna Salvetti (INSERM, France) and comprised Ad.TRE.ICP0 containing the HSV-1 strain 17 ICP0 gene under the control of the tetracycline-responsive (TRE) promoter, Ad.TRE.FXE containing a non-functional RING finger deletion of the HSV-1 strain 17 ICP0 gene, and Ad.CMV.rtTA encoding the reverse tetracycline transactivator under the control of the HCMV IE promoter. In these three recombinants, the transgene was inserted in place of the E1 gene in an E1/E3-deficient backbone.

For *β*-galactosidase staining, infected MRC-5 cells, either before or after de-repression, were fixed with 0.5 % glutaraldehyde in PBS and then overlaid with 5-bromo-4-chloro-3-indolyl *β*-d-galactopyranoside (X-gal) solution (0.01 % sodium deoxycholate, 0.02 % NP-40, 2 mM MgCl_2_, 1 mg X-gal ml^−1^, 5 mM potassium ferricyanide and 5 mM potassium ferrocyanide) and incubated at 37 °C for 6 h.

### ChIP assays.

Cell monolayers were fixed with 1 % formaldehyde for 10 min at 37 °C before removal into ice-cold PBS containing EDTA-free protease inhibitor cocktail (Roche) and 1 mM PMSF (Roche). Cell pellets (4×10^6^ cells) were resuspended in 1.2 ml lysis buffer [50 mM Tris/HCl (pH 8.0), 1 % SDS, 10 mM EDTA] containing protease inhibitors and incubated on ice for 10 min. The cell lysate was sonicated (Vibracell; Jencons Scientific Ltd) at 40 % output twice for 2 min each to shear the chromatin into a median size range of 500–1000 bp. Aliquots (250 μl) of cell lysate were diluted 10-fold in dilution buffer [20 mM Tris/HCl (pH 8.0), 1 % Triton X-100, 2 mM EDTA, 150 mM NaCl]. Each aliquot was pre-cleared with 100 μl protein A–Sepharose beads [0.5 g protein A–Sepharose (Sigma) in 5 ml dilution buffer containing 1 mg salmon sperm DNA ml^−1^, 1 mg BSA ml^−1^ and 0.02 % sodium azide] for 1 h at 4 °C. Volumes of chromatin (250 μl) were incubated overnight with anti-pan-acetylated histone H3 (acetyl K9 and K14) (06-599; Upstate Biotechnology), anti-histone H3 trimethyl K9 (ab8898; Abcam), anti-HP1-*α* (ab9057; Abcam), anti-C-terminal histone H3 (ab1791; Abcam), anti-pan-acetylated histone H4 (acetyl K4, K7, K11 and K15) (06-866; Upstate Biotechnology) or anti-pan-acetylated histone H4 (raised against chemically acetylated histone H4) (ab193; Abcam). Antibody–antigen complexes were isolated with protein A–Sepharose beads and eluted by incubation with 1 % SDS in 0.1 M NaHCO_3_. Eluted DNA–protein complexes were incubated with 0.2 M NaCl at 65 °C overnight to reverse the formaldehyde cross-linking and the DNA was purified by treatment with proteinase K followed by phenol/chloroform extraction. The DNA was isolated by ethanol precipitation following the addition of glycogen and tRNA as carrier.

### Standard PCR analyses.

The genomic region of interest was subjected to a maximum of 35 cycles of PCR amplification using AmpliTaq Gold Polymerase (Roche) using the following primer sets: ICP0: 5′-TATACCCCACGCCTTTCCCC-3′ [forward primer (FP), nt 2089–2070] and 5′-CCTTGTTCCGCTTCCCGGTA-3′ [reverse primer (RP), nt 1553–1572]; glycoprotein C (gC): 5′-GTTTTCCGAGGTTGTCGTGT-3′ (FP, nt 95830–95849) and 5′-GGTCTTCGGGACTAATGCCT-3′ (RP, nt 96088–96069); LAT promoter: 5′-CCCAGAGTCATTGTTTATGTGG-3′ (FP, nt 118519–118540): 5′-AGCAAAAACAGGCCACAGC-3′ (RP, nt 118759–118741). PCRs on input DNA and bound ChIP fractions were performed simultaneously and sampled after 20, 25 and 30 cycles. The PCR conditions were as follows: initial denaturation for 5 min at 95 °C, cycles of 1 min at 94 °C, 1 min at 55 °C (gC and LAT primers) or 65 °C (ICP0 primers) and 1 min at 72 °C, and a final elongation of 10 min at 72 °C. PCR products were resolved by agarose gel electrophoresis and analysed by Southern blot hybridization using radiolabelled probes specific for each amplicon.

### Quantitative real-time PCR.

Real-time PCR was carried out using a Rotor-Gene (Corbett Research) in triplicate for each reaction. The primer sets and Taqman probes employed for amplification of each virus promoter, given as HSV-1 sequence co-ordinates, were as follows: ICP0: nt 2072–2090 (FP), nt 2209–2139 (RP), nt 2115–2134 (probe); ICP4: nt 131522–131538 (FP), nt 131747–131730 (RP), nt 131700–131678 (probe); ICP27: nt 113238–113259 (FP), nt 113384–113367 (RP), nt 113328–113308 (probe); VP16: nt 105408–105436 (FP), nt 105712–105695 (RP), nt 105439–105462 (probe); gC: nt 95689–95707 (FP), nt 95800–95783 (RP), 95756–95777 (probe); LAT promoter: nt 118248–118267 (FP), nt 118374–118351 (RP), nt 118323–118303 (probe); LAT enhancer: nt 119314–119334 (FP), nt 119425–119405 (RP), nt 119336–119360 (probe). The transcription start sites for the HSV promoters were as follows: ICP0, nt 2115; ICP4, nt 131429; VP16, nt 105259; ICP27, nt 113596; gC, nt 96170; LAT, nt 118801. The following primers were also used: human GAPDH: nt 1725–1744 (FP), nt 1913–1894 (RP), nt 1749–1773 (probe) (relative to the transcription start site of nt 1874; GenBank accession no. AY340484); human tumour necrosis factor (TNF)-*α*: nt 1768–1789 (FP), nt 2262–2241 (RP), nt 1878–1904 (probe) (Nitsche *et al.*, 2001); human *γ*-globin: nt 1999–2020 (FP), nt 2185–2166 (RP), nt 2147–2121 (probe) (Bottardi *et al.*, 2003). Primers and HPLC-purified probes were manufactured by TIB-Molbiol. PCR products were quantified using a Rotor-Gene and associated software as the copy number per PCR, calculated from triplicate results from each PCR. A standard curve for each gene region was generated using dilutions of appropriate plasmids. The level of association of modified histones in the original chromatin sample was expressed as the ratio of immunoprecipitated DNA (IP-DNA) (bound) to the total amount of DNA in the chromatin sample (input). Fold changes in the association of histones in uninduced latently infected material in comparison with associations observed following induction of reactivation were calculated as the ratio of IP-DNA/input (induced) to IP-DNA/input (uninduced). The level of association of modified histones at the human GAPDH region was quantified and expressed as an IP-DNA/input ratio from which fold changes relative to uninduced chromatin were calculated. To normalize the fold changes against human GAPDH, each fold change occurring at a viral promoter was divided by the fold change occurring at GAPDH in the corresponding chromatin sample.

## RESULTS

### Establishment of quiescent infection in MRC-5 cells and reactivation by HSV-2 superinfection

**Fig. 1. f1:**
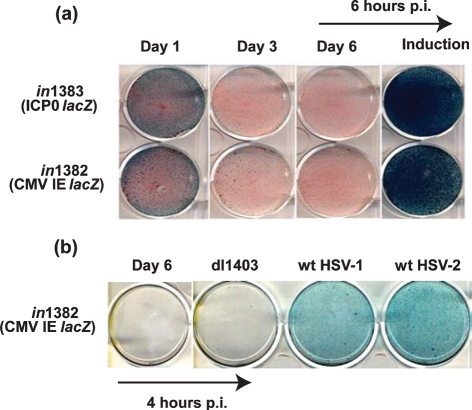
(a) *β*-Galactosidase staining of MRC-5 cells infected with *in*1382 or *in*1383 at 1, 3 and 6 days p.i. or at 6 days p.i. following induction by superinfection with HSV-2 strain 333 for 6 h. (b) *β*-Galactosidase staining of MRC-5 cells infected with *in*1382 at 6 days p.i. or at 6 days p.i. following superinfection with either HSV-1 ICP0 mutant dl1403, wild-type (wt) HSV-1 strain 17 or wt HSV-2 strain 333 for 4 h.

Previous studies have shown that infection of cells in culture with IE gene-deficient mutants of HSV-1 results in the establishment of a quiescent state (Harris & Preston, 1991; Preston & Nicholl, 1997; Samaniego *et al.*, 1998; Minaker *et al.*, 2005). Quiescent genomes remain functional, as reactivation can be induced efficiently by provision of ICP0 *in trans* following superinfection with HSV or recombinant adenovirus (Zhu *et al.*, 1990; Preston & Nicholl, 1997; Hobbs *et al.*, 2001; Minaker *et al.*, 2005). In order to facilitate analyses of the chromatin status of HSV DNA during quiescence and following the induction of reactivation, we used an MRC-5 cell-based system to generate material for ChIP analyses. Quiescent infection was established by infecting MRC-5 cells with replication-defective HSV-1 mutants carrying mutations in the virion transactivator VP16, ICP0 and *ts*ICP4, and the reporter gene *lacZ* under the control of either the HCMV IE promoter (*in*1382) or the ICP0 promoter (*in*1383). Infection of cells at an m.o.i. of 1 at the non-permissive temperature of 39 °C resulted in transient LacZ expression from the HCMV IE or ICP0 promoter with shut-off evident at day 3 through to day 6 p.i. (Fig. 1a[Fig f1]). Induction of reporter gene expression 6 h after HSV-2 superinfection demonstrated the retention of functional and responsive virus genomes. The inability of the HSV-1 ICP0 deletion mutant dl1403 to de-repress and activate HCMV IE promoter-driven *lacZ* expression from MRC-5 cells harbouring quiescent *in*1382 genomes indicated an essential requirement for ICP0 in genome de-repression (Fig. 1b[Fig f1]).

The fate of input DNA was evaluated by real-time PCR analysis of DNA extracted from cells infected at an m.o.i. of 1 with *in*1382 sampled at 4 and 24 h p.i. and at day 6 p.i., either before or after HSV-2 superinfection. HSV-1-specific ICP0 primers were utilized and, in order to prevent genome amplification, superinfected monolayers were cultured in the presence of PAA. A rapid decrease in levels of virus DNA was evident at early times p.i., with the virus genome copy number falling from 3572 to 240 copies per cell between 4 and 24 h p.i. relative to GAPDH. This rapid decrease in DNA with high DNA input loads is consistent with previous studies of related replication-defective mutants (Harris & Preston, 1991; Jamieson *et al.*, 1995). By 6 days p.i., the virus DNA load had fallen to 169±26.8 (±sem) copies per cell (*n*=4) and had not changed appreciably 6 h following genome de-repression (106±25.9). The relative stability of HSV DNA copy number before and after de-repression validated the utility of this experimental system in studies of the chromatin status of quiescent HSV genomes.

### Changes in histone modifications associated with the HSV-1 genome during quiescence and de-repression mediated by HSV-2 superinfection

**Fig. 2. f2:**
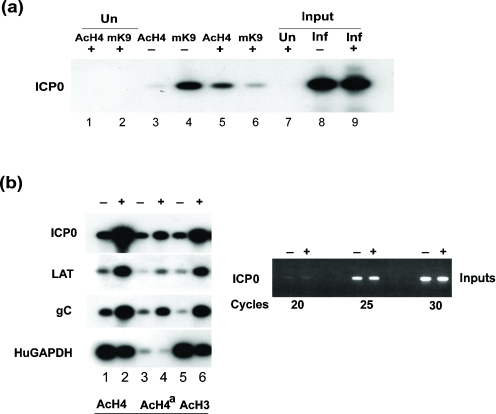
(a) ChIP analysis of MRC-5 cells, uninfected (Un) or latently infected with *in*1382 and, at 6 days p.i., either superinfected (+) or not (−) with wt HSV-2 strain 333 for 6 h in the presence of PAA. Antibodies used were anti-acetyl histone H4 (AcH4) or anti-trimethyl K9 histone H3 (mK9). PCRs were performed using primers specific for the HSV-1 ICP0 promoter. (b) Left panel: ChIP analysis of MRC-5 cells latently infected with *in*1382 and, at 6 days p.i., either superinfected (+) or not (−) with wt HSV-2 strain 333 for 6 h in the presence of PAA. Antibodies used were anti-acetyl histone H4 (AcH4), chemically acetylated H4 (AcH4^a^) or anti-acetyl histone H3 (acetyl K9 and K14) (AcH3). PCRs were performed using primer sets to the ICP0, LAT, gC or GAPDH promoters. Right panel: PCRs using ICP0 primers on input chromatin.

To examine the nature of modified histones associated with HSV DNA during quiescence and de-repression following HSV-2 superinfection, histone modifications were examined using ChIP assays. Our initial attention focused on the association at the ICP0 promoter of pan-acetylated histone H4 (AcH4), a marker of transcriptionally active chromatin, and trimethylation of lysine 9 of histone H3 (TMK9H3), a marker of repressed chromatin (Jenuwein & Allis, 2001). An increase in association of AcH4 was observed at the ICP0 promoter 6 h after HSV-2 superinfection relative to quiescent genomes (Fig. 2a[Fig f2], lanes 3 and 5). Conversely, during quiescence, there was a clear association of the repressive TMK9H3 marker and a detectable decrease in this modified histone at the ICP0 promoter following genome de-repression (Fig. 2a[Fig f2], lanes 4 and 6). These results indicated that acquisition of a repressed state during quiescence of HSV in MRC-5 cells is associated with a repressed histone marker (TMK9H3) and that de-repression following superinfection is associated with histone H4 acetylation at the ICP0 promoter, a representative IE gene promoter. In order to determine whether the observed increased association of acetylated histones at the ICP0 promoter following de-repression reflected changes occurring at other genomic loci, we examined the gC late promoter and the LAT promoter. ChIP analyses revealed an increased association of AcH4 with antibodies raised against either H4 acetylated at K4, K7, K11 and K15 (AcH4) (Fig. 2b[Fig f2], lanes 1 and 2), chemically acetylated H4 (Fig. 2b[Fig f2], lanes 3 and 4) or pan-acetylated histone H3 (AcH3; Fig. 2b[Fig f2], lanes 5 and 6). Although differences in immunoprecipitation efficiencies were observed with these antibodies, in all cases an increased association of acetylated histones at virus promoters was evident following de-repression. The results shown in Fig. 2(b) (right panel) confirmed that equivalent amounts of input chromatin from latently infected or superinfected cells were analysed. No increase in association of acetylated histones was observed at the human GAPDH promoter following superinfection, suggesting that the acetylation status of this transcriptionally active housekeeping promoter is not altered by the de-repressive activity of the superinfecting virus.

**Fig. 3. f3:**
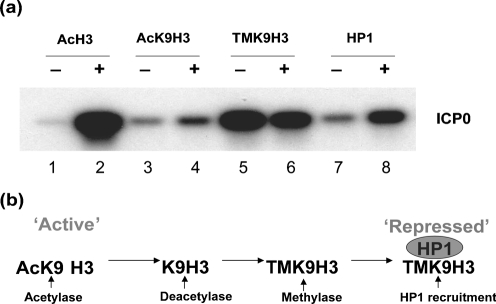
(a) ChIP analysis of MRC-5 cells latently infected with *in*1382 and, at 6 days p.i., either superinfected (+) or not (−) with wt HSV-2 strain 333 for 6 h in the presence of PAA. Antibodies used were anti-acetyl histone H3 (acetyl K9 and K14) (AcH3), anti-acetyl K9 of histone H3 (AcK9H3), anti-trimethyl K9 histone H3 (TMK9H3) and anti-HP1-*α* (HP1). PCRs were performed using HSV-1 primers to the ICP0 promoter. (b) Model of sequential repression of chromatin by loss of acetylation markers, gain of methylation and recruitment of HP1 to trimethylated histone H3.

**Fig. 4. f4:**
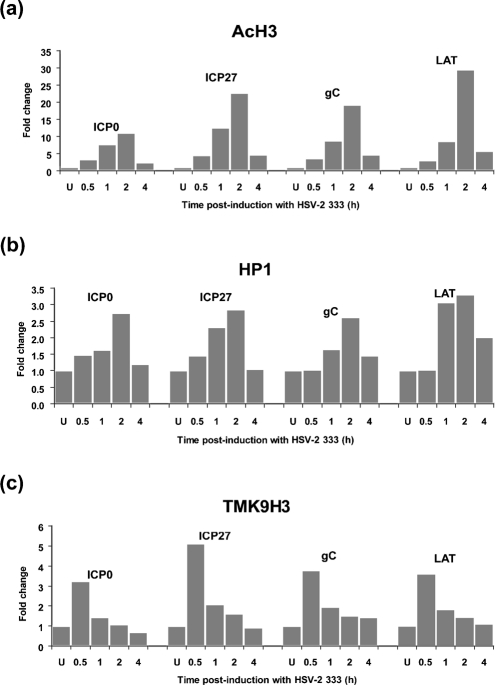
ChIP analysis using quantitative real-time PCR. MRC-5 cells were latently infected with *in*1382 and, at 6 days p.i., either superinfected or not with wt HSV-2 strain 333 for 0.5, 1, 2 or 4 h. Antibodies used were (a) anti-acetyl histone H3 (acetyl K9 and K14) (AcH3), (b) anti-HP1-*α* (HP1) or (c) anti-trimethyl K9 histone H3 (TMK9H3). PCRs were performed using primer sets to the ICP0, ICP27, gC or LAT promoter. Promoter copy number was quantified by real-time PCR in triplicate and the mean value used to calculate the IP-DNA/input ratio. This value was then expressed as a fold change relative to uninduced material. Fold changes were normalized to GAPDH as described in Methods. Histograms show fold changes in the level of AcH3, HP1 and TMK9H3 at promoters against time post-superinfection with HSV-2. The data shown are representative of three independent experiments.

The observation of TMK9H3 association at the ICP0 promoter during quiescence (Fig. 2a[Fig f2], lane 4) is consistent with virus genomes adopting a heterochromatic state of silencing, as has been reported previously in studies of HCMV latency (Murphy *et al.*, 2002; Reeves *et al.*, 2005). As TMK9H3 binds heterochromatic protein 1 (HP1) (Bannister *et al.*, 2001), we next determined the association of HP1 at the ICP0 promoter during quiescence and following de-repression (Fig. 3). Consistent with our earlier observations, an increase was observed in the association of acetylated histone H3 (AcH3) or acetyl K9 of histone H3 (AcK9H3) (Fig. 3a[Fig f3], lanes 1–4) and a modest decrease in association of TMK9H3 (Fig. 3a[Fig f3], lanes 5 and 6) at the ICP0 promoter following HSV-2 superinfection. Surprisingly, although an association of HP1 with the ICP0 promoter was detected during quiescence, an enrichment of HP1 was observed 6 h following HSV-2 superinfection (Fig. 3, lanes 7 and 8). Examination of a range of virus promoters (ICP0, ICP27, gC and LAT) by real-time PCR at various times after superinfection revealed a marked ∼10–28-fold change in AcH3 bound to virus promoters 2 h after superinfection, consistent with genome de-repression (Fig. 4a[Fig f4]). An increased association of HP1 was also observed at all promoters, with a maximal ∼2.5–3-fold change occurring 2 h post-superinfection (Fig. 4b[Fig f4]). This recruitment of HP1 was preceded by a transient enrichment of TMK9H3 at 0.5 h post-superinfection with a maximal ∼3.2–5-fold change observed compared with uninduced cells (Fig. 4c[Fig f4]). TMK9H3 and HP1 are generally considered to represent marks of transcriptionally repressed chromatin; it is therefore likely that their association with virus promoters represents an early response to superinfection with HSV-2 rather than de-repression mediated by ICP0 alone. It was therefore of interest to determine the impact of ICP0 expression on both the association of modified histones and HP1 recruitment to virus promoters in isolation using adenovirus vectors expressing ICP0.

### De-repression mediated by a recombinant adenovirus expressing ICP0 is associated with an enrichment of acetylated histones at virus promoters but not HP1

**Fig. 5. f5:**
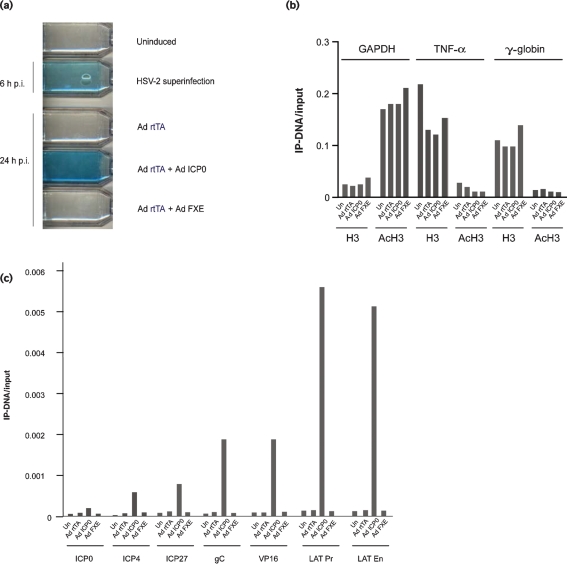
(a) *β*-Galactosidase staining of MRC-5 cells latently infected (uninduced) with *in*1382 at 6 days p.i. or superinfected with HSV-2, Ad.CMV.rtTA (Ad rtTA) alone or Ad rtTA with either Ad.TRE.ICP0 (Ad ICP0) or Ad.TRE.FXE (Ad FXE) in the presence of doxycycline. ChIP analyses of MRC-5 cells latently infected with *in*1382, either uninduced (Un) or 24 h after superinfection with Ad rtTA, Ad ICP0, or Ad FXE. ChIPs were performed with anti-acetyl histone H3 (AcH3) or anti-C-terminal histone H3-specific (H3) antibodies. (b, c) Real-time PCRs were performed with primer sets to the promoters of GAPDH, TNF-*α* and *γ*-globin (b) and to a range of virus promoters as indicated (c). Promoter copy number was quantified by PCR in triplicate and the mean values used to calculate the IP-DNA/input ratios. The data shown are representative of at least two independent experiments.

The ability of an adenovirus vector carrying the ICP0 gene under the control of the doxycycline/rtTA-inducible promoter (Ad.TRE.ICP0) to de-repress quiescent HSV-1 genomes was evaluated by superinfecting MRC-5 cells 6 days after infection with *in*1382 (Fig. 5a[Fig f5]). In comparison with uninduced MRC-5 cells, which exhibited no detectable HCMV IE promoter-driven *β*-galactosidase expression from resident *in*1382 genomes, superinfection with a mixture of Ad.CMV.rtTA and Ad.TRE.ICP0 in the presence of doxycycline resulted in genome de-repression and detection of *β*-galactosidase. The lack of detectable *β*-galactosidase following superinfection with either Ad.CMV.rtTA or Ad.TRE.FXE, carrying a non-functional copy of ICP0 deleted in the RING finger domain, confirmed the essential requirement for this functionally important region of ICP0 in genome de-repression.

Chromatin harvested from either uninduced cells or cells superinfected with a mixture of Ad.CMV.rtTA and Ad.TRE.ICP0 were next subjected to ChIP analyses. First, the impact of adenovirus superinfection on the IP-DNA/input ratios of cellular GAPDH, TNF-*α* and *γ*-globin was examined. Delivery of functional ICP0 did not impact the level of AcH3 at either transcriptionally active (GAPDH) or transcriptionally silent (TNF-*α*, *γ*-globin) genes and had no impact on nucleosome occupancy as assessed using a histone H3 C-terminal-specific antibody (Fig. 5b[Fig f5]). Consistent with previous genome-wide studies of nucleosome occupancy and histone acetylation (Pokholok *et al.*, 2005) the transcriptionally active GAPDH promoter displayed a decreased nucleosome occupancy and elevated association with AcH3 in comparison with the transcriptionally repressed TNF-*α* or *γ*-globin genes. In contrast to the inability of ICP0 to increase the association of AcH3 with the cellular genes examined, ICP0 expression resulted in a marked increase in the IP-DNA/input ratios across a range of virus promoters (Fig. 5c[Fig f5]).

**Fig. 6. f6:**
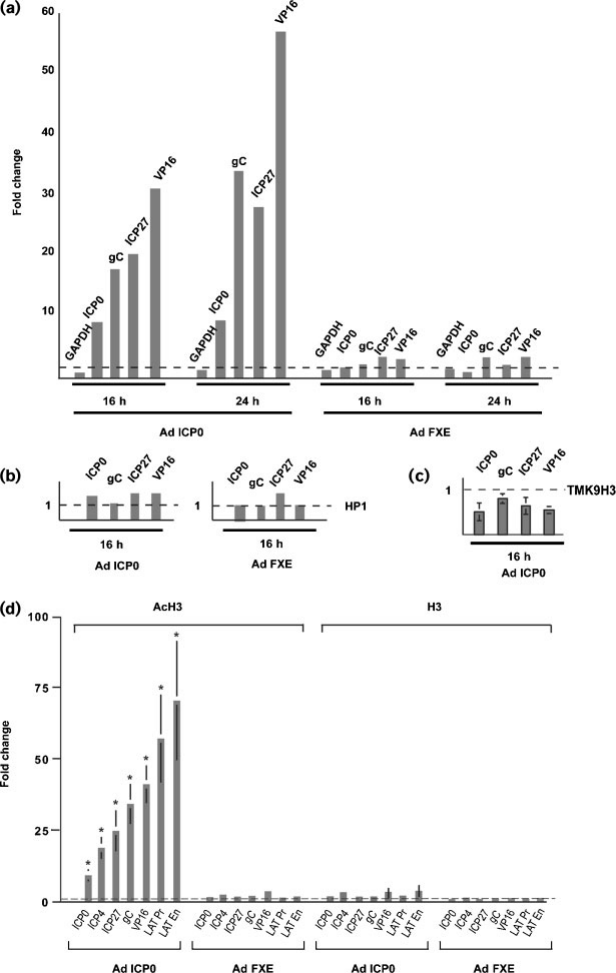
ChIP analyses of MRC-5 cells latently infected with *in*1382 and at 6 days p.i., superinfected for 16 or 24 h with Ad.CMV.rtTA (Ad rtTA) and either Ad.TRE.ICP0 (Ad ICP0) or Ad.TRE.FXE (Ad FXE) in the presence of doxycycline. (a–c) ChIPs were performed with anti-acetyl histone H3 (K9 and K14) (a), anti-HP1-*α* (b) or anti-trimethyl K9 histone H3 (TMK9H3) (c). Real-time PCRs were performed with primer sets to the promoters of GAPDH, ICP0, gC, ICP27 or VP16. Promoter copy number was quantified by PCR in triplicate and the mean value used to calculate the IP-DNA/input ratio. This value was then expressed as a fold change relative to uninduced material (dashed line=1). The data in (c) represent mean fold changes from three independent experiments (±sem). (d) ChIP analyses of MRC-5 cells latently infected with *in*1382 and superinfected at 6 days p.i. for 24 h with Ad rtTA and either Ad ICP0 or Ad FXE. ChIPs were performed with anti-acetyl histone H3 (AcH3) or anti-C-terminal histone H3 (H3)-specific antibodies. Promoter copy number was quantified by PCR in triplicate and the mean value used to calculate the IP-DNA/input ratio. This value was then expressed as a fold change relative to uninduced material (dashed line=1). Fold changes were normalized to GAPDH as described in Methods. Histograms show the mean fold changes from five independent experiments (±sem). *, *P*<0.05 versus uninduced material using a two-tailed Student's *t*-test.

The delivery of functional ICP0 to quiescently infected cells resulted in an enrichment of AcH3 at all virus promoters examined and, with the exception of the ICP0 promoter, the fold change in acetylation increased between 16 and 24 h following adenovirus transduction (Fig. 6a[Fig f6]). The enrichment of AcH3 at virus promoters was dependent on the RING finger of ICP0, as transduction of quiescently infected cells with Ad.TRE.FXE did not result in an increased association of AcH3 at virus promoters, consistent with the inability of this adenovirus vector to induce *β*-galactosidase expression from resident *in*1382 genomes. In contrast to the modest but reproducible enrichment of HP1 at virus promoters following HSV-2 superinfection, delivery of ICP0 using an adenovirus vector did not result in an obvious enrichment of HP1 at virus promoters (Fig. 6b[Fig f6]). However, a reduction in association of TMK9H3 was observed at virus promoters following ICP0-mediated de-repression (Fig. 6c[Fig f6]). The overwhelming conclusions from these experiments are that: (i) there is the existence of a direct correlation between histone acetylation at virus promoters and HSV genome de-repression; (ii) there is a dependency on an intact ICP0 RING finger for this effect; (iii) delivery of ICP0 in isolation does not lead to an obvious recruitment of HP1; and (iv) de-repression is associated with a reproducible decrease in association of TMK9H3.

A striking observation was the lack of uniformity of AcH3 association at different virus promoters following Ad.TRE.ICP0 infection. Thus, at 24 h after delivery of Ad.TRE.ICP0, the highest fold changes (>25-fold) were observed at the VP16, ICP27 and gC promoters with only an 8-fold change observed at the ICP0 promoter (Fig. 6a[Fig f6]). Fig. 6(d) shows the mean fold change (*n*=5) in AcH3 associated with a range of virus promoters and confirmed the non-uniform pattern of AcH3 enrichment following Ad.TRE.ICP0 superinfection. In order to determine whether the differences observed were influenced by the degree of histone occupancy at the various promoters, we performed a further ChIP assay using an antibody specific for the C terminus of histone H3, which detects both modified and unmodified forms of histone H3 (Fig. 6d[Fig f6]). This antibody revealed only minor differences in the fold change of histone H3 associated with the various test promoters following Ad.TRE.ICP0 or Ad.TRE.FXE superinfection, suggesting that nucleosome occupancy at these promoters is relatively uniform and is unlikely to account for the relatively large differences in the fold changes of AcH3 at HSV promoters following ICP0-mediated de-repression.

## DISCUSSION

Previous studies have used replication-defective mutants of HSV-1 to establish quiescent infection of non-neuronal cells and have shown that virus genomes adopt a quiescent state that can efficiently be de-repressed by the provision of ICP0 *in trans* (reviewed by Preston, 2000). In this study, we examined the association of modified histones with repressed and de-repressed genomes following either superinfection with HSV-2 or delivery of ICP0 using an adenovirus vector. Consistent with previous studies, infection of MRC-5 cells with *in*1382 resulted in transient HCMV IE promoter-driven LacZ expression. The inability to detect LacZ at later times after infection indicated the establishment of a repressed state that could be reversed following superinfection with HSV-2 or Ad.TRE.ICP0.

The inability to de-repress quiescent genomes following superinfection with the HSV-1 ICP0 mutant dl1403 or an adenovirus containing a non-functional ICP0 gene is consistent with earlier studies demonstrating a crucial role for ICP0 in genome de-repression (reviewed by Preston, 2000). The precise mechanism by which ICP0 mediates its effect is unclear. However the observation that ICP0 expression converts quiescent genomes from a repressed state characterized by an under-representation of acetylated histone H3 and H4 to a transcriptionally permissive form of chromatin enriched with acetylated histones suggests that a key response to ICP0 is the reversal of histone-mediated gene silencing.

Our initial studies focused on the association of modified histones at the ICP0 promoter. ChIP analyses revealed a relative lack of association of AcH3 and AcH4 at this promoter during quiescence, with a marked increase in association of these acetylated histones following HSV-mediated de-repression. Conversely, during quiescence, there was a strong association of the repressive TMK9H3 marker and a decrease in association of this modified histone at the ICP0 promoter following genome de-repression. Examination of the ICP27, gC and LAT promoters revealed a similar pattern of association of acetylated and methylated histone markers during both quiescence and de-repression, suggesting a global silencing mechanism associated with an enrichment of repressive histone markers at promoters, independent of their kinetic class. Examination of HP1, which is recruited to TMK9H3 and is a characteristic marker of heterochromatic gene silencing during HCMV latency (Murphy *et al.*, 2002; Reeves *et al.*, 2005), revealed an association of HP1 with representative virus promoters during quiescence. However, de-repression mediated by HSV-2 superinfection resulted in an increase in association of HP1, with a 2.5–3-fold enrichment observed 2 h after superinfection. De-repression mediated by superinfection with Ad.TRE.ICP0 resulted in an enrichment of AcH3 at all virus promoters examined, but did not result in an increased association of HP1, suggesting that the recruitment of HP1 to a range of virus promoters following HSV-2 superinfection could be the consequence of delivery and/or expression of HSV-2-encoded gene products. Such a view is not without precedent, as recent studies of HCMV major IE promoter regulation have shown that IE86-mediated autorepression is the result of IE86-mediated changes in chromatin structure of the viral major IE promoter characterized by HP1 recruitment (Reeves *et al.*, 2006).

The fold change in association of AcH3 following Ad.TRE.ICP0-mediated de-repression consistently revealed promoter-specific variation, with the greatest changes (>50-fold) being observed at the LAT promoter and enhancer regions. ChIP analyses using an antibody specific to the C terminus of histone H3 as a measure of nucleosome occupancy revealed little variability in nucleosome occupancy at the various HSV promoters. This observation suggests that acetylation of histone H3 in response to ICP0 expression is more efficiently targeted to regulatory regions of LAT than other representative virus promoters. Consistent with our observations for AcH3, examination of the association of AcH4 at the LAT promoter and enhancer following ICP0-mediated de-repression has revealed 22- and 17-fold changes, respectively (unpublished observations), and experiments are in progress to evaluate the pattern of acetylation of this histone at distinct virus promoters following de-repression mediated by ICP0.

Previous *in vivo* studies have revealed that, during latency, HSV DNA is retained in a repressed chromatinized state characterized by the association of hypoacetylated histones (Kubat *et al.*, 2004a, b) and that LATs may function to facilitate maintenance of a repressed chromatinized genome (Wang *et al.*, 2005). Furthermore, following ganglionic explantation, genome de-repression is linked to an enrichment of acetylated histones at lytic cycle promoters and a decrease in LAT enhancer histone acetylation and LAT RNA abundance (Amelio *et al.*, 2006). The decrease in histone acetylation at the LAT enhancer following explant culture of trigeminal ganglia reported by Amelio *et al.* (2006) contrasts with the high level of acetylation at the LAT regulatory region observed following ICP0-mediated de-repression observed in the current study. The most likely explanation for this apparently contradictory result is that analyses of sensory ganglia at early times post-explant (1–2 h) facilitates an examination of primary changes in the acetylation of histones at LAT regulatory regions prior to the expression of ICP0. In the current study, which utilized a non-neuronal *in vitro* latency model system, the acetylation of histones at virus promoters was dependent on ICP0 expression and may therefore reflect events that occur at later stages of virus reactivation following the expression of ICP0. The increased acetylation observed at the LAT promoter/enhancer in response to ICP0 protein expression could function to activate LAT transcription in order to antagonize lytic phase transcription and provide an additional checkpoint in the regulation of reactivation.

Our studies have utilized an *in vitro* model system to examine the nature of modified histones associated with virus genomes during quiescence and have revealed that, in the absence of virus gene expression, input genomes are effectively silenced and associated with histone markers characteristic of inactive chromatin. These results accord with recent data reporting an association of rapid gene silencing and the formation of inactive chromatin of HSV amplicon-based vectors (Suzuki *et al.*, 2006). The observation that ICP0-mediated de-repression of quiescent genomes is associated with enrichment of active histone markers at virus promoters is consistent with reports demonstrating an interaction of ICP0 with histone deacetylases (HDACs) (Lomonte *et al.*, 2004) and its ability to dissociate HDAC 1 and 2 from the CoREST/REST complex (Gu *et al.*, 2005). It would therefore appear that the ICP0 gene product plays a key role in antagonizing genomic silencing in the *in vitro* model system described. Whether ICP0 plays a similar crucial role at the earliest stages of reactivation from latency in sensory neurones is less clear. Unlike non-neuronal model systems of HSV quiescence, which are dependent on ICP0 for genome de-repression, reactivation from sensory neurones can be mediated by a wide variety of external stimuli. Furthermore, the observation that ICP0-deficient mutants can initiate virus gene expression in a proportion (<0.1 %) of latently infected neurones *in vivo* suggests that an ICP0-independent mechanism of de-repression operates in this subset of neurones (Thompson & Sawtell, 2006). Further studies are clearly warranted to determine the requirement for ICP0 in genome de-repression and induction of reactivation in the majority of latently infected neurones that are refractory to external stimuli.
